# Malarial Hæmaturia

**Published:** 1872-07

**Authors:** Charles P. Gordon

**Affiliations:** Dalton, Georgia


### MALARIAL HÆMATURIA.

BY CHARLES P. GORDON, M.D., DALTON, GEORGIA.

Please find inclosed the following report of a case of malarial hæmaturia. I write from memory, having made no notes of the case at the time:

Andrew Pickens Calhoun, aged twenty years, very tall in statue, ordinarily robust and vigorous in constitution, but now reduced by successive attacks of intermittent fever, having resided recently in the swamps of Arkansas and Louisiana—a young gentleman of brilliant intellect and manly bearing, the descendant of distinguished ancestry—came under my charge March 7th, 1872, suffering with parotitis, complicated with violent bilious remittent fever, attended with excessive nausea, and vomiting very similar to the black vomit of yellow fever, with an icterode complexion; urine scanty and exceedingly high-colored, accompanied with pain and difficulty in voiding it. A mercurial cathartic was prescribed, followed by the sulphate of quinine, with the hypodermic injection of sulphate of morphine to procure rest at night, which promptly arrested the violent symptoms, and in eight or ten days, the patient was entirely convalescent.

Riding out early on the morning of the 18th of March, he "felt pain in the right side," and was compelled to return home immediately, when he was seized with violent congestion of the lungs, attended with rapid and laborious breathing, and the expectoration of dark, congested blood, with a return of all the biliary symptoms—heavily-coated tongue, excessive nausea, and vomiting of altered blood, jaundiced complexion, with difficulty in voiding urine, almost amounting to strangury. A mercurial cathartic was again prescribed, with large doses of sulphate quinine, with partial relief of congestive symptoms, and finally with intense relief of thoracic congestion—nausea and vomiting continuing, however, with such obstinacy as to render it impossible to retain food and medicines. The hypodermic injection of sulphate of morphine was again resorted to, with great temporary relief, and decided hope of recovery.

The fifth day from his relapse, with an aggravation of all his symptoms, was observed an entirely new phenomenon—that of *hœmaturia*. Whether the urine had contained blood previously or not, is not known, no microscopic examination having been made; it was now perceptibly mixed with blood. Henceforward the case progressed more uniformly, giving, sometimes, hopes of amelioration and improvement, again worse—attended all the while with nausea and occasional vomiting, with "strange sensations" (to use the language of the patient) about the head, and occasionally a wild and violent paroxysm of delirium, with muscular tremors, bordering on convulsions, which, continuing a half hour, were followed by profound coma, when the patient would again awake rational. The intellect was remarkably clear during his illness, with the exception of the paroxysms of deliriums above mentioned, when he would seem to be upon the verge of an *uremic eclampsia.* He died April 4th, nineteen days from his relapse, by asthenia.

I would venture an opinion, from the observation of this case: that malarial hæmaturia is a violent form of congestive fever, with involvement of the renal organs in active congestion and inflammation, attended with all the danger and fatality consequent upon an obliteration of their proper function, and the retention in the blood of those elements excreted by the kidneys. As we have in congestive fever the cerebral, the thoracic, the abdominal varieties, I would add another—the *renal variety,* in which the congestive tendency is to the kidneys, with all the pernicious results following malarial hæmaturia.

As to the treatment of this disease, in its early stages, as in all the other forms of malarial congestive fever, I would rely upon the sulphate of quinine as the sheet-anchor, with mercurial cathartics to relieve biliary derangement—carefully abstaining from ptyalism, from the fact, that should the violence of the disease be arrested by the anti-periodic treatment, the patient may have to pass through a long and tedious attack of disease, resulting from the lesion sustained by the kidneys, and a retention in the blood of those poisonous elements which those organs, in a state of disease, fail to eliminate. In the latter stages, I would rely upon a supporting treatment, with the mineral acids and the muriated tincture of iron.

---

Treatment of Dissecting Wounds.—Dr. John H. Beach, Coldwater, Michigan, (*Detroit Review of Medicine and Pharmacy*), having been poisoned by a dissecting wound, was cured through the influence of about fifteen grains of concentrated carbolic acid internally, in about eleven hours of medication. Diluting a solution of carbolic acid so that he could swallow it without much irritation of the fauces, he began taking doses equivalent to about twenty drops of the saturated solution, every half hour.—*Medical Record.*

